# Radical oxygen species: an important breakthrough point for botanical drugs to regulate oxidative stress and treat the disorder of glycolipid metabolism

**DOI:** 10.3389/fphar.2023.1166178

**Published:** 2023-05-12

**Authors:** Maocai Luo, Yuhong Zheng, Shiyun Tang, Linsen Gu, Yi Zhu, Rongtao Ying, Yufei Liu, Jianli Ma, Ruixin Guo, Peiyang Gao, Chuantao Zhang

**Affiliations:** ^1^ Department of Respiratory Medicine, Hospital of Chengdu University of Traditional Chinese Medicine, Chengdu, China; ^2^ GCP Center, Hospital of Chengdu University of Traditional Chinese Medicine, Chengdu, China; ^3^ Department of Critical Care Medicine, Hospital of Chengdu University of Traditional Chinese Medicine, Chengdu, China

**Keywords:** botanical drugs, glycolipid metabolic diseases, oxidative stress, radical oxygen species, mitochondria function, nicotinamide adenine dinucleotide phosphate hydrogen oxidase, signaling pathways

## Abstract

**Background:** The incidence of glycolipid metabolic diseases is extremely high worldwide, which greatly hinders people’s life expectancy and patients’ quality of life. Oxidative stress (OS) aggravates the development of diseases in glycolipid metabolism. Radical oxygen species (ROS) is a key factor in the signal transduction of OS, which can regulate cell apoptosis and contribute to inflammation. Currently, chemotherapies are the main method to treat disorders of glycolipid metabolism, but this can lead to drug resistance and damage to normal organs. Botanical drugs are an important source of new drugs. They are widely found in nature with availability, high practicality, and low cost. There is increasing evidence that herbal medicine has definite therapeutic effects on glycolipid metabolic diseases.

**Objective:** This study aims to provide a valuable method for the treatment of glycolipid metabolic diseases with botanical drugs from the perspective of ROS regulation by botanical drugs and to further promote the development of effective drugs for the clinical treatment of glycolipid metabolic diseases.

**Methods:** Using herb*, plant medicine, Chinese herbal medicine, phytochemicals, natural medicine, phytomedicine, plant extract, botanical drug, ROS, oxygen free radicals, oxygen radical, oxidizing agent, glucose and lipid metabolism, saccharometabolism, glycometabolism, lipid metabolism, blood glucose, lipoprotein, triglyceride, fatty liver, atherosclerosis, obesity, diabetes, dysglycemia, NAFLD, and DM as keywords or subject terms, relevant literature was retrieved from Web of Science and PubMed databases from 2013 to 2022 and was summarized.

**Results:** Botanical drugs can regulate ROS by regulating mitochondrial function, endoplasmic reticulum, phosphatidylinositol 3 kinase (PI3K)/protein kinase B (AKT), erythroid 2-related factor 2 (Nrf-2), nuclear factor κB (NF-κB), and other signaling pathways to improve OS and treat glucolipid metabolic diseases.

**Conclusion:** The regulation of ROS by botanical drugs is multi-mechanism and multifaceted. Both cell studies and animal experiments have demonstrated the effectiveness of botanical drugs in the treatment of glycolipid metabolic diseases by regulating ROS. However, studies on safety need to be further improved, and more studies are needed to support the clinical application of botanical drugs.

## 1 Introduction

Glycolipid metabolic diseases are a large group of diseases characterized by disorders of glycolipid metabolism, including many diseases, such as diabetes mellitus (DM), non-alcoholic fatty liver disease (NAFLD), obesity, and atherosclerosis (AS)*.* This kind of disease tends to be chronic, which affects the extension of human life to a certain extent and brings a heavy burden to human health cause. According to the statistics of “The International DM Federation,” there will be 536.6 million DM patients worldwide in 2021 (Sun et al., 2022). Studies have found that about 1/4 of the global population suffers from NAFLD (Huang et al., 2021). In addition, the incidence of obesity, AS, hyperlipidemia, and other diseases presents a gradually increasing trend (Barquera et al., 2015; Bluher, 2019; Pirillo et al., 2021). Oxidative stress (OS) plays an important role in the pathogenesis of glycolipid metabolic diseases.

OS is the role of REDOX signal transduction widely existing in organisms (Sies, 2015), which is the imbalance between oxidizing free radicals and antioxidants. It is as broad and important as body PH regulation (Sies et al., 2017). Radical oxygen species (ROS) is a general term for a large group of oxidants extended from molecular oxygen, including superoxide (O^2−^) and hydrogen peroxide (H_2_O_2_) (Sies et al., 2022). There are two main sources of ROS in the body: one is from enzymes and the other is from mitochondria. The enzymes that generate ROS include NADPH oxidase (NOX), lipoxygenase (LOX), and nitric synthase (NOS), among which the most important is NADPH oxidase (Brown and Griendling, 2015). Mitochondrial ROS, as a by-product of the ATP production process, increases during anoxia or mitochondrial dysfunction (Shadel and Horvath, 2015). As a key factor of signal transduction in OS, ROS plays a role in regulating cell proliferation, inflammation, and body aging (Schieber and Chandel, 2014) and progressing diseases related to glucose and lipid metabolism.

## 2 Association of ROS with common glycolipid metabolic diseases

### 2.1 ROS and DM

DM is a group of complex metabolic diseases, often manifested by the abnormal metabolism of carbohydrates, fats, and proteins due to the insufficient action of insulin and pancreatic islet B cells (Farmer and Fox, 2011). In the OS mode, ROS and DM are closely related. DM promotes ROS production by reducing intracellular antioxidant levels (Vezza et al., 2021), and ROS accelerates the dysregulation of glucose metabolism and tissue damage through a series of signal transduction.

In DM, ROS can be produced by NADPH oxidase, ER stress (Leenders et al., 2021), mitochondrial stress, and abnormal fatty acid metabolism (Drose and Brandt, 2012; Qiu and Zhang, 2019; Zhou L. et al., 2021; Zhou Y. et al., 2021). Under pathological stimulation, glucose glycolysis produces a large number of ROS, resulting in elevated blood sugar and forming a vicious cycle of hyperglycemia-OS (Qiu and Zhang, 2019).

The accumulation of ROS will damage islet B cells and weaken their function (Blesia et al., 2021). ROS inhibited the expression of insulin promoter factor 1 (Pdx-1) by activating the JNK pathway (Kajimoto and Kaneto, 2004), thus reducing insulin production. In addition, ROS can induce apoptosis of islet B cells by, for example, regulating intracellular C_a_^2+^ concentration (Gier et al., 2009) and consuming heparan sulfate proteoglycan (Dhounchak et al., 2021). In addition, ROS can further cause mitochondrial dysfunction, resulting in reduced proliferation and differentiation of islet B cells (Nahdi et al., 2017). ROS can cause not only islet B-cell dysfunction, but also insulin resistance (IR). On the one hand, ROS can induce insufficient glucose uptake and trigger IR (Bhattacharya et al., 2017) by inhibiting GLUT-4 expression (Yaribeygi et al., 2020). On the other hand, ROS can also impair insulin sensitivity and promote the occurrence of IR by interfering with signaling pathways or downregulating the concentration of signaling molecules (Cheng et al., 2021). In addition, ROS influence on mitochondrial function can lead to IR production (Yaribeygi et al., 2020). Therefore, ROS and DM have a mutually reinforcing relationship.

### 2.2 ROS and NAFLD

NAFLD covers a wide spectrum of liver injury pathologic spectrum, from general steatosis and steatohepatitis to liver fibrosis and cirrhosis, and it is a typical disorder of lipid metabolism. Currently, there is a “multiple shocks” hypothesis related to the pathogenesis of NAFLD, and OS is a major factor in liver injury and this disease progression (Takaki et al., 2013; Friedman et al., 2018). Abnormal lipid metabolism further promotes ROS production, whereas ROS accumulation aggravates OS, leading to the further development of NAFLD (Mansouri et al., 2018), forming a vicious cycle.

ROS in NAFLD can be produced by mitochondria, and mitochondrial fatty acid oxidative overload leads to increased mitochondrial ROS production. However, the mitochondrial electron transport chain (ETC) complex cannot be upregulated in a coordinated manner, which leads to ROS overproduction (Begriche et al., 2013). In addition, mitochondrial flavoenzymes (including pyruvate dehydrogenase, α-ketoglutarate dehydrogenase, glycerol phosphate, and electron transfer flavoprotein) in mitochondria are considered a major source of ROS (Chen et al., 2020). ER stress is also an important source of ROS in NAFLD. The increase in ROS in the ER is mainly due to the excessive utilization of reduced GSH to reduce the oxidized unfolded protein response (UPR) (Zeeshan et al., 2016) and the increase in UPR-mediated ROS production by the upregulation of CHOP activity (Fuchs et al., 2017).

On the one hand, excessive production of ROS can affect mitochondrial function and lead to abnormal fatty acid oxidation. On the other hand, ROS can damage macromolecules and lead to the production of toxic substances. In addition, ROS and gut microbiota interact to promote liver inflammation. All these contribute to the development of NAFLD (Arroyave-Ospina et al., 2021).

### 2.3 ROS and AS

AS is a chronic multifactorial inflammatory disease of arterial blood vessels and is the main cause of cardiovascular disease morbidity and mortality. ROS accompanies every step in the development of AS, including the expression of adhesion molecules, stimulation of vascular smooth muscle proliferation and migration, endothelial cell apoptosis, lipid oxidation, and activation of matrix metalloproteinases (Park and Oh, 2011). Matrix metalloproteinases degraded the fibrous wall of AS plaques and the basement membrane of endothelial cells. This leads to the physical destruction and shedding of plaques, ultimately leading to microvascular destruction, microbleeds, and thrombosis (Kattoor et al., 2017).

The generation of ROS in AS mainly depends on various oxidases (Forstermann et al., 2017), among which NADPH oxidases are the main ROS generators in the cardiovascular system, and the deletion of its two subtypes, NOX1 and NOX2, has been confirmed to be related to the reduction of AS in mice (Kattoor et al., 2017). In macrophages, NOX activity plays a role in endothelial adhesion molecule expression, monocyte infiltration, and vascular smooth muscle cell VSMC proliferation (Vendrov et al., 2007). In addition, elevated levels of other enzymes, such AS superoxide anions, were associated with the acceleration of AS (Madamanchi and Runge, 2007). 5/12/15-lipoxygenases are correlated with the occurrence of AS (Cervantes Gracia et al., 2017), which induce NOX activation in vascular endothelial cells and lead to OS. The end product, leukotriene, also has a pro-inflammatory effect.

ROS has multiple effects on the development of AS, including oxidative modification of lipids and DNA, endothelial dysfunction, and the stability of plaque fiber caps (Batty et al., 2022). ROS accumulation increased the content of ox-LDL and the lesion area of AS (Shih et al., 2000). ROS can damage nuclear and mitochondrial DNA (mtDNA) damage, accelerate the development of AS, and increase susceptibility to AS (Yu et al., 2013; Shah et al., 2018). It can also cause endothelial cell inflammation and damage endothelial function (Li and Mehta, 2000). In addition, ROS can promote the production of interleukin to mediate the recruitment of macrophages (Libby, 2017). All these functions play an important role in the development of AS.

### 2.4 ROS and other glycolipid metabolic diseases

#### 2.4.1 Obesity

Obesity, which is usually defined as being severely over recommended weight levels, is associated with excess accumulation of fat. In obesity, excessive fat accumulation and IR promote ROS production, whereas moderate caloric restriction increases antioxidant activity (Kanikowska et al., 2021). The accumulation of ROS further promotes the occurrence of obesity and its development (Martinez-Martinez and Cachofeiro, 2022).

#### 2.4.2 Hyperlipidemia

Hyperlipidemia is characterized by increased blood lipid levels, including increased LDL and VLDL, and decreased high-density lipoprotein particles. Hypercholesterolemia leads to ROS production; decreases the antioxidant activities of GSH, SOD, and catalase enzymes leading to homocysteine autoxidation; and weakens the intracellular PI3K/AKT pathway due to the decrease in PTP enzymes, JAK/STAT, and PARP. It accelerates endothelial dysfunction (Jamwal and Sharma, 2018). However, the high level of ROS further increases the level of ox-LDL and the severity of hyperlipidemia.

In addition, hypoglycemia (Ceriello et al., 2013) and metabolic syndrome (Ando and Fujita, 2009) are also related to ROS. The overall performance is mutual promotion and mutual influence.

**FIGURE 1:**
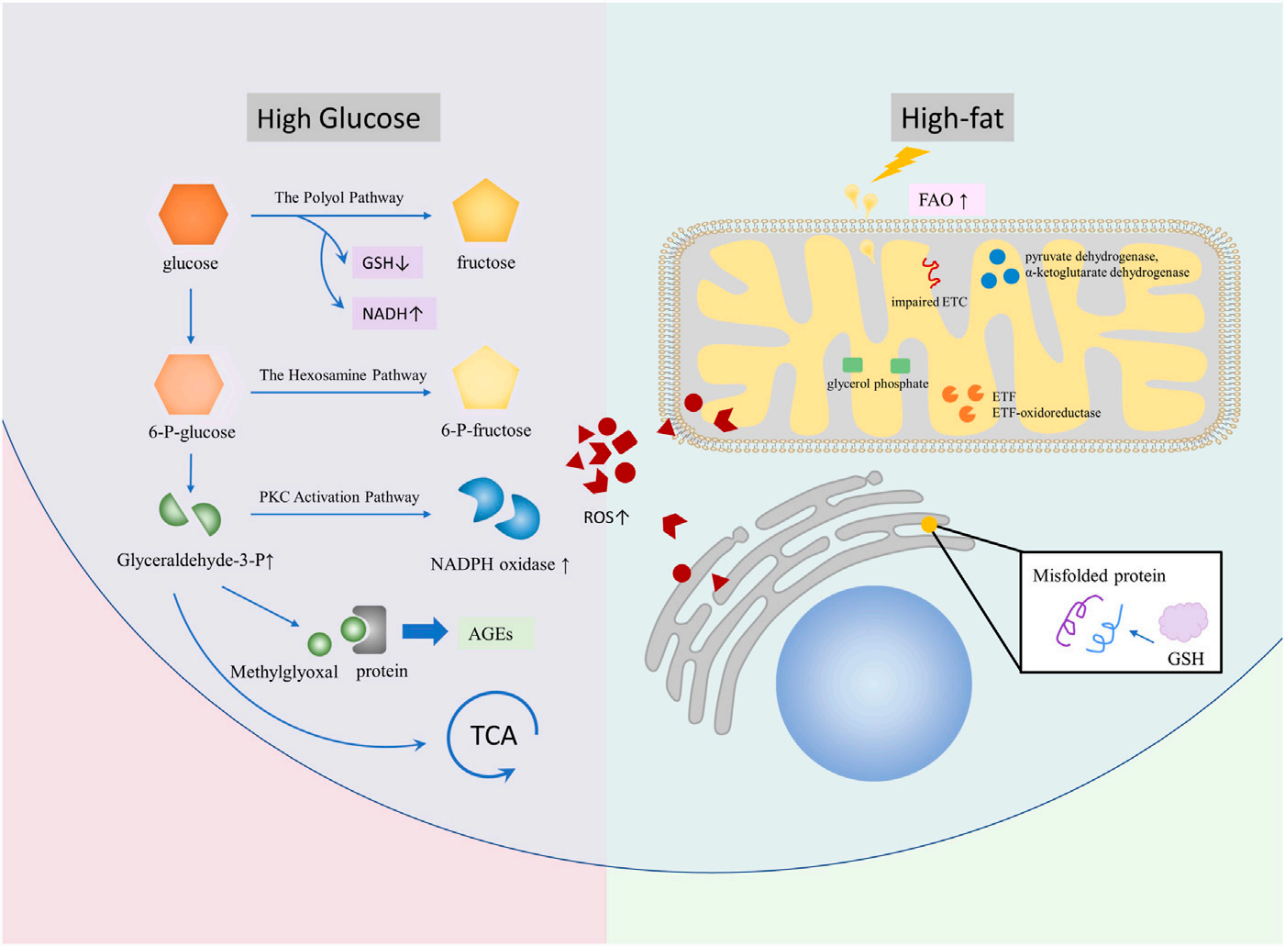
Diseases, such as DM, NAFLD, and AS, lead to glycolipid metabolism disorder and promote ROS production. In a high-sugar environment, ROS can generate more ROS via branching off pathways, such as the polyol pathway, the hexosamine pathway, the PKC activation pathway, and AGE, on the basis of glycolysis. In the high-fat environment, ROS is mainly caused by excessive oxidative stress of mitochondria and ER caused by increased FAO. A large number of ROS are involved in glycolipid metabolism, which causes complications.

In conclusion, ROS is produced in glycolipid metabolic diseases and plays an important role in the occurrence, development, and deterioration of such diseases (Figure 1). In order to improve the development process of glucose and lipid metabolism diseases, special treatment for ROS in OS is still an emerging direction.

Some previous drugs for the treatment of glycolipid metabolic diseases by inhibiting OS are metformin (Wang et al., 2017; Apostolova et al., 2020), sodium-glucose co-transporter-2 inhibitors (Trnovska et al., 2021), and statins for the treatment of hypercholesterolemia (Nagila et al., 2009; Carnevale et al., 2010). However, ROS as an entry point for the treatment of glycolipid metabolic diseases is still a new direction. In recent years, nano-targeted drugs targeting ROS have been developed to antagonize or eliminate ROS (Yoshitomi and Nagasaki, 2014; Saifi et al., 2021). However, nanomedicine has not been fully transformed into clinical medication due to the differences in production quality, insufficient large-scale production plans, and the need to evaluate drug safety (Zhang Z. et al., 2022). These reasons make us find safer drugs with excellent anti-OS effects, and botanical drugs give us a new choice.

**FIGURE 2:**
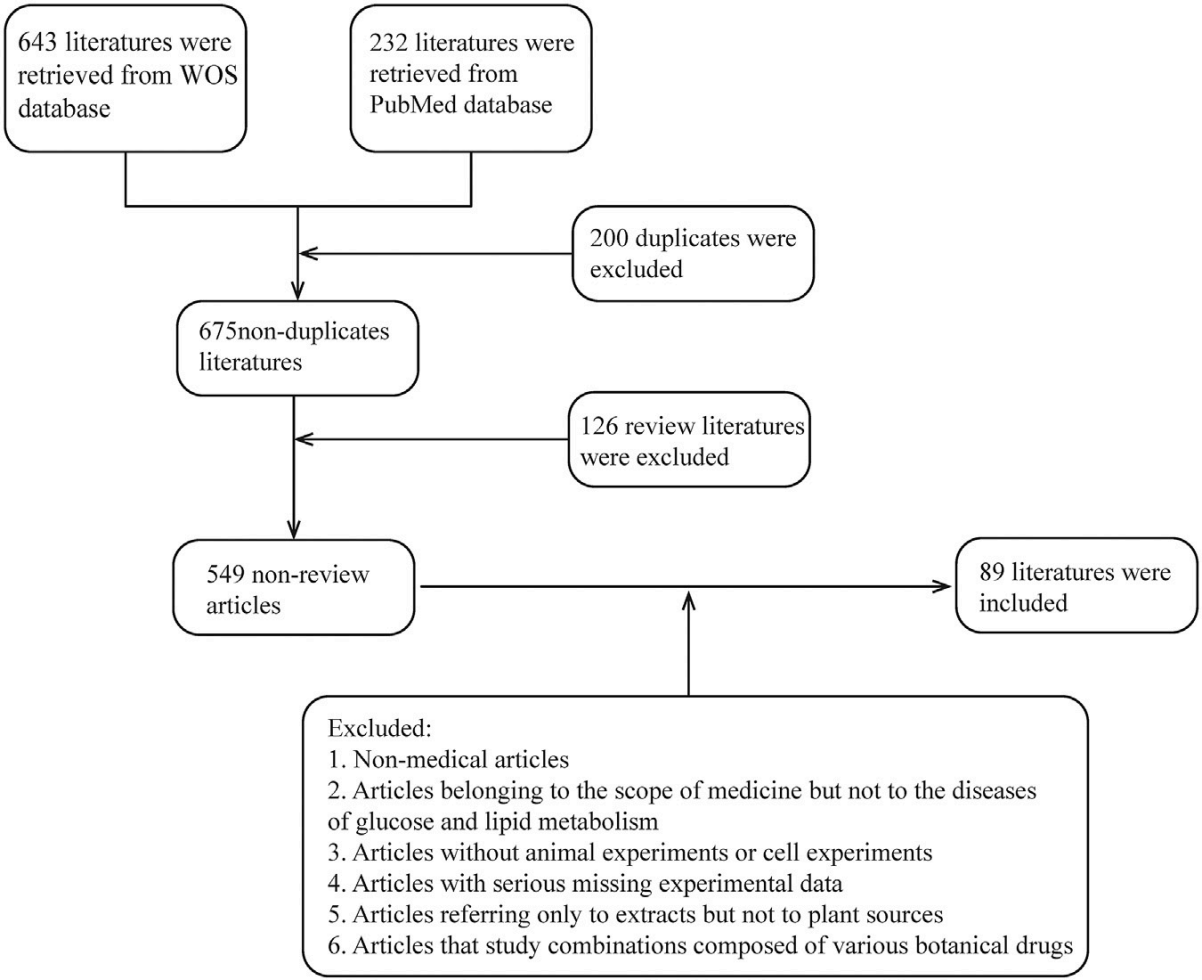
Literature screening process; review literature is automatically identified by the database.

To determine how botanical drugs modulate ROS in diseases of glycolipid metabolic, herb, plant medicine, Chinese herbal medicine, phytochemicals, natural medicine, phytomedicine, plant extract, botanical drug, ROS, oxygen free radicals, oxygen radical, oxidizing agent, glucose and lipid metabolism, saccharometabolism, glycometabolism, lipid metabolism, blood glucose, lipoprotein, and triglyceride, fatty “liver”, “atherosclerosis”, “obesity OR diabetes”, “dysglycemia”, “NAFLD”, and “DM” were used as keywords or subject headings to search for relevant articles in the Web of Science and PubMed databases from 2013 to 2022. A total of 680 articles were retrieved, of which 126 review articles were excluded and 554 articles were selected. Subsequently, we screened the articles and excluded those with the following conditions: 1) non-medical articles; 2) articles belonging to the scope of medicine but not to the diseases of glucose and lipid metabolism; 3) articles without animal or cell experiments; 4) articles with serious missing experimental data; 5) articles referring only to extracts but not to plant sources; and 6) articles that study combinations composed of various botanical drugs (e.g., decoctions). Finally, 89 articles were included, involving 81 kinds of botanical drugs, which summarized the species and genera of plants and the extraction methods involved in plant extraction. Unfortunately, due to the incomplete information provided by the author, five species of plants have not been found (Table 1) (the screening process is shown in Figure 2).

**TABLE 1:** Botanical drugs information table.

Plant source	Family	Used part	Extract	Extraction method	Qualitative phytochemical analysis	Ref.
*Alisma plantago-aquatica* subsp. *orientale*	Alismataceae	Rhizome	Alisol A 24-acetate		Triterpenoids	Wu et al. (2018)
*Alnus firma*	Betulaceae	Leaves	Ethanolic extract	Ethanol extraction	Phenolic, flavonoids, proanthocyanidin	Choi et al. (2018)
*Alpinia officinarum Hance*	Zingiberaceae	Rhizome	DPH5	Extract by petroleum ether and ethyl acetate	Diarylheptanoid component	Zhang et al. (2022a)
*Amaranthus viridis*	Amaranthaceae	Leaves		Moringa methanol extracts	Kaempferol, quercetin, catechin, gallic acid, caffeic acid, p-coumaric acid, vanillin, ferulic acid, protocatechuic acid, cinnamic acid, and epicatechin	Omodanisi et al. (2017)
*Angelica gigas Nakai*	Umbelliferae	Root	Ligustilide		Phthalide derivative	Choi et al. (2018)
*Annickia polycarpa*	Annickia	Stem bark		Water extraction	Saponins, reducing sugars, phenolic compounds, alkaloids, flavonoids	Lartey et al. (2021)
*Anoectochilus roxburghii*	Orchidaceae		ARP		Six monosaccharides	Liu et al. (2017)
*Anoectochilus roxburghii* Wall. Lindl.	Orchidaceae		Kinsenoside			Liu et al. (2016)
*Antrodia Cinnamomea*	Polyporales			Concentration		Yen et al. (2020)
*Artemisia capillaris*	Asteraceae		Capillin		Polyacetylene	Li et al. (2021a)
*Artemisia caruifolia*	Asteraceae	Leaves		Water extraction	Polyphenols, flavonoids, condensed tannins	Sekiou et al. (2021)
*Astragalus mongholicus*	Fabaceae		Astragaloside IV			Zhu et al. (2019)
*Calotropis procera*	Apocynaceae	Latex	Laticifer proteins	Centrifugal method	Abundant proteins	De Oliveira et al. (2021)
*Cannabis sativa*	Cannabaceae		Cannabidiol			Jiang et al. (2021)
*Cassia auriculata Linn*	Leguminosae	Flower		Ethanolic extract of *C. auriculata* flowers		Vijayaraj et al. (2013)
*Catharanthus roseus*	Apocynaceae		Vindoline		Indole alkaloid	Goboza et al. (2019)
*Cistanche tubulosa*	Orobanchaceae		Echinacoside			Kong et al. (2018)
*Cnidium monnieri*	Apiaceae	Rhizome	Ligustilide		Phthalide derivative	Choi et al. (2018)
*Crassocephalum crepidioides*	Asteraceae	Aerial parts		Methanol extraction	Phenolic and flavonoids	Bahar et al. (2017)
*Crataegus aronia*	Rosaceae	Aerial parts		Water extraction		Mani et al. (2022)
*Curcuma*	Zingiberaceae	rhizome	Curcumin		Polyphenolic compound	Cao et al. (2022)
*Dendrobium huoshanense*	Orchidaceae	Stems	DHP	Water extraction	Polysaccharide	Fan et al. (2020)
*Dendrobium officinale*	Orchidaceae					Han et al. (2021)
*Dillenia indica*	Dilleniaceae	Leaves	DI-HET	Extract by n-hexane, ethyl acetate, hydroethanolic		Poornima et al. (2022)
*Echinodorus grandiflorus*	Alismataceae	Leaves				Gasparotto et al. (2019)
*Enicostemma littorale* Blume	Gentianaceae	Whole plant	Betulin, swertiamarin, enicoflavine, swertisin	EL MeOH ext	Triterpenoid sapogenin, secoiridoid glycoside, flavonoid, and gentiocrucine	Srivastava et al. (2016)
*Epimedium*	Berberidaceae		Icariside II		Flavonoid	Zhang et al. (2020)
*Eugenia jambolana*	Myrtaceae	Seeds		Extract by petroleum ether, aqueous acetone, EtOAc, and n-BuOH	Hyperglycemia	Liu et al. (2018)
*Galega officinalis*	Fabaceae	Aboveground part		Ethanol extraction	Non-alkaloid fraction	Hachkova et al. (2021)
*Ganoderma lucidum*	Ganodermataceae	Fruiting bodies	FYGL	Extract by ethanol and acetic acid	Proteoglycan	Liang et al. (2020)
*Ginkgo biloba*	Ginkgoaceae		Bilobalide			Su et al. (2022)
*Ginkgo biloba*	Ginkgoaceae		*Ginkgo biloba* extract			Chang et al. (2021)
*Gynura bicolor*	Ginkgoaceae	Leaves	GBEE	Ethanol extraction		Hsieh et al. (2020)
*Herba Epimedii*	Berberidaceae		Icariside II			Li et al. (2022)
*Herba Erigerontis*	Aristolochiaceae		Scutellarin			Fu et al. (2019)
*Hibiscus sabdariffa*	Malvaceae					Herranz-Lopez et al. (2020)
*Homalium zeylanicum*		Calyx	Quercetin		Polyphenol	Herranz-Lopez et al. (2020)
*Hydrangea paniculata*	Hydrangeaceae					Sen et al., 2019
*Hypoxis hemerocallidea*	Amaryllidaceae	Corm		Hypoxis hemerocallidea aqueous extract		Oguntibeju et al. (2016)
*Ilex chinensis Sims*	Aquifoliaceae		Coumarin glycosides			Deng et al. (2021)
*Inonotus obliquus*	Hymenochaetaceae	Sclerotium	Phelligridin D	Extract by petroleum ether and ethyl acetate	Phenolic compound	Li et al. (2021b)
*Juglans regia*	Juglandaceae	Husk		Ethanol extraction		Fang et al. (2022)
*Lannea coromandelica*	Anacardiaceae	Bark	LCBE		Polyphenolic compounds	Alam et al. (2017)
*Laurus nobilis Linn*		Leaves		Ethanol extraction	Methyl eugenol, kaempferol rutinoside/isomer, gallic acid	Bourebaba et al. (2021)
*Laurus nobilis* Linn.	Lauraceae		*Laurus nobilis*	*Laurus nobilis* ethanolic extract		Bourebaba et al. (2021)
*Ligusticum chuanxiong* Hort.	Umbelliferae	Rhizome	*Ligusticum chuanxiong* 19	LC ethanolic extract	17b-Estradiol, phenylephrine hydrochloride, acetylcholine hydrochloride, sodium nitroprusside, pentobarbital sodium, ferulic acid, and tetramethylpyrazine	Li et al. (2014)
*Lindera obtusiloba*	Lauraceae	Branch	LOE	Ethanol extraction	Hyperin, isoquercitrin, guaijaverin, avicularin, and quercitrin	Ihm et al. (2021)
*Lithospermum erythrorhizon* Sieb. et Zucc	Boraginaceae		Shikonin			Huang et al. (2015)
*Momordica charantia*	Cucurbitaceae	Leaves		Water extraction	Polyphenol and flavonoids	Hsu et al. (2021)
*Morinda citrifolia*	Rubiaceae	Pulp, seeds		Extract by n-butanol		Ishibashi et al. (2017)
*Moringa oleifera*	Moringaceae	Leaves	Ascorbic acid, rutin, quercetin, and catechin	Concentrated methanol extract from crape myrtle leaves	Flavonoids, phenols, saponins, tannins, alkaloids, terpenoids, and steroid	Salvamani et al. (2016)
*Nepeta angustifolia* C. Y. Wu	Lamiaceae	Areal parts		Ethanol extraction	Oleanolic acid, betulinic acid, and ursolic acid	Huang et al. (2020)
*Ophiocordyceps sinensis*	Ophiocordycipitaceae		Cordycepin			Ku et al. (2021)
*Padina pavonia*				Extract by dichloromethane	Terpenoids	Germoush et al. (2020)
*Paeonia lactiflora* Pall.	Paeoniaceae		Paeoniflorin			Yang et al. (2016)
*Paeonia suffruticosa* Andr.	Paeoniaceae	Root	Moutan	Moutan ethanolic extract		Zhang et al. (2014)
*Panax ginseng* C. A. Meyer	Araliaceae		Panax notoginseng saponins	PNS	Ginsenoside Rb1	Fan et al. (2016)
*Parkia biglobosa*	Fabaceae	Seeds		N-Hexane, butanol extraction	Protein	Ogunyinka et al. (2019)
*Picrorhiza kurroa*	Plantaginaceae		Apocynin			Gimenes et al. (2018)
*Premna herbacea*	Lamiaceae	Leaves	Isoverbascoside	Methanol extraction		Kashyap et al. (2021)
*Prosopis Strombulifera*	Fabaceae					Quesada et al. (2021)
*Prunella vulgaris* Linn.	Labiatae		*P. vulgaris*	*P. vulgaris* ethanol extract		Park et al. (2013)
*Pueraria lobata* Willd. Ohwi	Leguminosae	Root	Puerarin, daidzin, and daidzein	An ethanol extract from kudzu		Gao et al. (2016)
*Romina strawberry*		Pulp		Methanol extraction	Polyphenol and flavonoids	Forbes-Hernández et al. (2017)
*Rubia cordifolia*, *Rubia tinctorum* L.	Rubiaceae	Root	Purpurin		Anthraquinones	Nam et al. (2019)
*Rubus amabilis*	Rosaceae	Stems		Acetone extraction	Procyanidins	Sun et al. (2020)
*Rubus coreanus* Miq.	Rosaceae		Rubus coreanus	URFE		Kim et al. (2013)
*Rumex dentatus* L.	Polygonaceae	Acrial part	RDE	Extract by methylene chloride, ethyl acetate, and n-butanol	Phenolic compounds	Elsayed et al. (2020)
*Salvia miltiorrhiza*	Lamiaceae			Water extraction	Dihydrotanshinone I, cryptotanshinone, tanshinone I, dihydrotanshinone I, and tanshinoneⅡA	Qin et al. (2016)
*Salvia miltiorrhiza* Bunge	Lamiaceae		Danshenol A		Abietane-type diterpenoid	Zhao et al. (2017a)
*Salvia plebeia*	Lamiaceae		Hispidulin	DMSO solution extraction	Flavone	Qin et al. (2016)
*Scutellaria baicalensis*	Lamiaceae	Root	Baicalin		Flavonoids	Chen et al. (2019)
*Scutellaria baicalensis* Georgi	Labiatae		Baicalin, wogonin		Baicalin, baicalein, and wogonin	Ku and Bae (2015)
*Silybum marianum* Linn. Gaertn	Compositae		Silymarin	Diluted with DMSO	DMSO, propylene glycol, and normal saline	Khazim et al. (2013)
*Smallanthus sonchifolius*	Asteraceae	Leaves		Acetone extraction	Non-alkaloid fraction	Hachkova et al. (2021)
*Sophora flavescens*	Fabaceae		Oxymatrine		Quinolizidine alkaloid	Jin et al. (2021a)
*Syzygium aqueum*	Myrtaceae	Leaves		Methanol extraction	Glucosides, flavonols myricetin, quercetin, and proanthocyanidins	Mahmoud et al. (2021a)
*Syzygium jambos*	Myrtaceae	Bark		Water extraction	Flavonoids, tannins, chalcones, phloroglucinol, and triterpenoids	Mahmoud et al. (2021b)
*Tessaria Absinthioides*	Asteraceae			Water extraction		Quesada et al. (2021)
*Tinospora sinensis*	Menispermaceae			Water extraction	Organic acids, phenolic acids, procyanidins, flavonoids, and oxylipins	Banerjee et al. (2020)
*Toxicodendron vernicifluum*	Anacardiaceae		IBF-R	Water extraction	Fisetin	Hoang et al. (2021)
*Tribulus terrester* Linn.	Zygophyllaceae		*Tribulus terrestris*	Aqueous extracts of *Tribulus terrestris*		Jiang et al. (2016)
*Trigonella foenum-graecum*	Fabaceae	Seeds	Orientin, isoorientin, vitexin, and isovitexin		Flavonoid glycosides	Luan et al. (2018)
*Trigonella foenum-graecum*	Fabaceae	Seeds	Polyphenol stilbenes		Polyphenol	Li et al. (2018)
*Tripterygium wilfordii* Hook. F	Celastraceae		Celastrol	Diluted with DMSO		Jiang et al. (2016)
*Zingiber officinale*	Zingiberaceae			Steam extraction, ethanol extraction	6-Gingerol	Lee et al. (2021)
	Ginkgoaceae			*Ginkgo biloba* extract		Tsai et al. (2013)

**NOTE:** ARP: *Anoectochilus roxburghii* polysaccharide, DPH5: 1,7-diphenyl-4E-en-3-heptanone, DHP: *Dendrobium huoshanense* C. Z. Tang et S. J. Cheng polysaccharide, DI-HET: *D. indica* hydroethanolic extract, FYGL: Fudan-Yueyang *G. lucidum*, Fisetin: a flavanol compound majorly found in IBF-R, GBEE: *G. bicolor* ether extract, IBF-R: lyophilized to obtain dried *R. verniciflua* extract, LCBE: *Lannea coromandelica* (Houtt.) Merr. Bark extract, LOE: *Lindera obtusiloba* extract, RDE: *R. dentatus* extract, Six monosaccharides: L-rhamnose, L-arabinose, D-xylose, D-mannose, D-glucose, and D-galactose.

(Botanical drugs through the following URL and the relationship between species: https://www.plantplus.cn/cn/sp/Homalium%20zeylanicum; https://www.catalogueoflife.org/; http://mpns.kew.org/mpns-portal/; http://www.plantsoftheworldonline.org).

In the following, we will explain the regulatory effects of botanical drugs on ROS regulation from the direct generation and scavenging of ROS, the regulation of related pathways, and the tolerance to reduce the adverse consequences caused by excessive ROS.

## 3 Multi-mechanism regulation of ROS by botanical drugs

### 3.1 Direct modulation of ROS production and scavenging by botanical drugs

**FIGURE 3:**
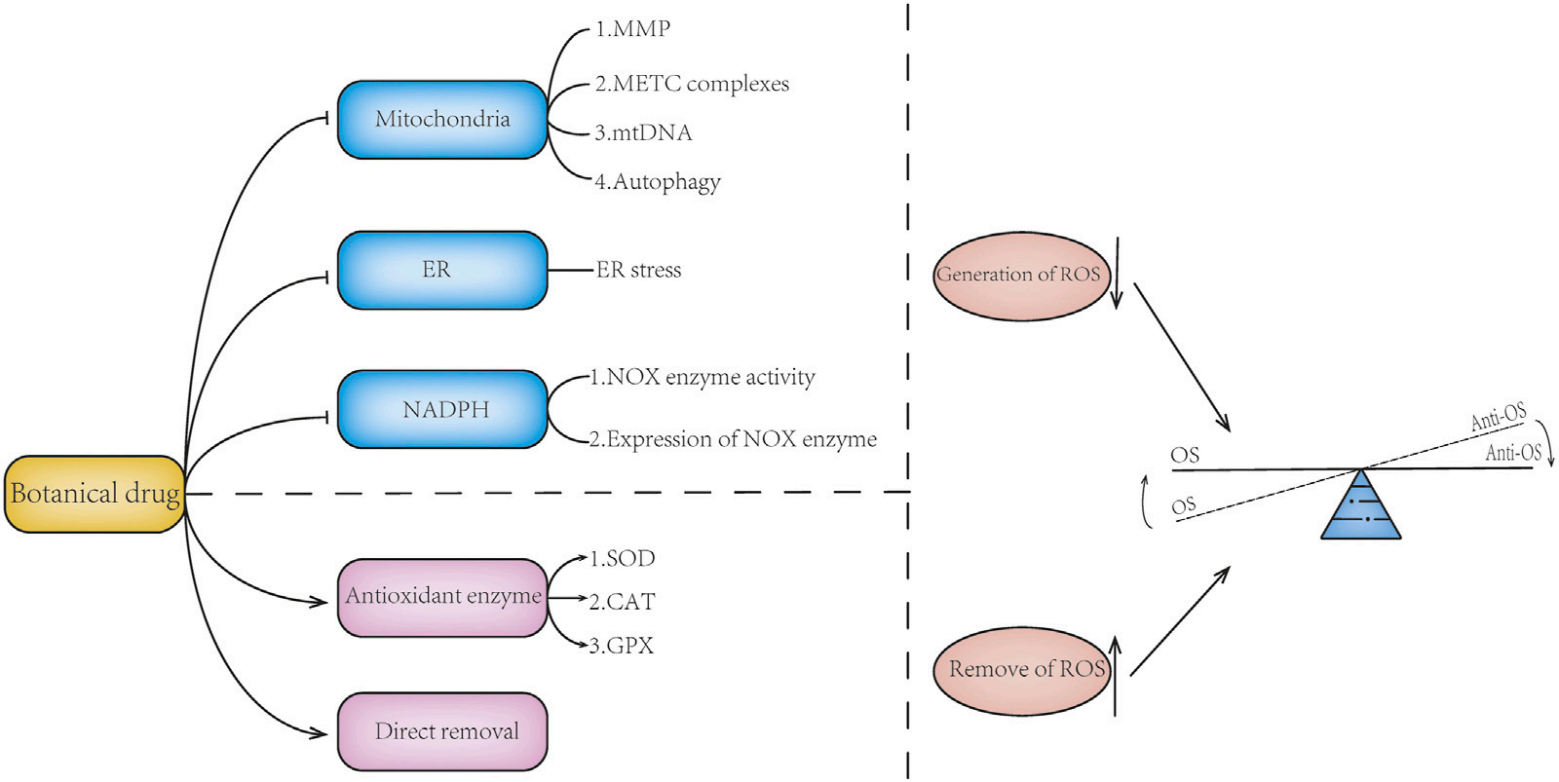
Botanical drugs directly modulate ROS generation or scavenging. Botanical drugs can regulate the balance of oxidative stress by reducing the generation of ROS and promoting the removal of ROS. Botanical drugs can reduce ROS production by regulating mitochondrial function, maintaining mitochondrial membrane potential, regulating the expression of mitochondrial ETC complex, protecting mitochondrial DNA, regulating mitophagy, improving ER stress, and reducing NOX enzymes. There are also botanical drugs that can reduce the accumulation of ROS and regulate the balance of oxidative stress by regulating the expression and activity of antioxidant enzymes and scavenging ROS. At the same time, some of the mechanisms of botanicals are not reflected in the aforementioned; for example, botanical drugs can inhibit the expression of LOX enzyme and reduce ROS production.

Mitochondria, endoplasmic reticulum, and related oxidases are important sources of ROS. Botanical drugs can directly regulate the production of ROS by regulating mitochondria, NADPH oxidase, and endoplasmic reticulum stress. Botanical drugs can also play a role by regulating the activity and expression of anti-OS enzymes (Table 2) (Figure 3).

**TABLE 2:** Research on ROS production directly regulated by botanical drugs.

Disease	Source	Animals/cell lines	Dose	Duration	Detail	Ref.
AS	*Salvia plebeia*	HUVECs	0.1, 1, 10 μM	24 h	Maintain the MMP	Qin et al. (2016)
AS	*Astragalus mongholicus*	HUVECs	10, 20, 50 μM	1 h	Inhibit NADPH oxidase activity	Zhu et al. (2019)
AS	*Lindera obtusiloba*	ApoE^−/−^ mice	100 mg/kg	20 weeks	Inhibit NADPH oxidase activity	Ihm et al. (2021)
AS	*Prosopis Strombulifera Tessaria Absinthioides*	VSMCs	2.5–40 µg/ml	24–48 h	Inhibit NADPH oxidase activity	Quesada et al. (2021)
AS	*Salvia miltiorrhiza* Bunge	HUVECs	10 nM	1 h	Inhibit TNF-α-induced NOX4 expression Inhibit TNF-α-induced NF-κB activation Inhibit ICAM-1 expression	Zhao et al. (2017a)
AS	*Pueraria lobata* Willd. Ohwi	HUVECs	1, 5, 10, 25 g/ml	48 h	Maintain the MMP	Gao et al. (2016)
AS	*Tripterygium wilfordii* Hook. F.	Macrophages	25–200 nmol/L	24 h	Scavenger receptor LOX-1	Gu et al. (2013)
		C57BL/6J mice	1, 2 mg/kg		Suppression of NF-κB pathway	
DM	*Laurus nobilis* Linn.	HepG2 cells	1 μg/ml	24 h	Regulate mitochondrial phosphorylation	Bourebaba et al. (2021)
					Maintain the MMP	
					Increase cell sensitivity to insulin	
DM	*Tinospora sinensis*	Wister rats	100–400 mg/kg	4 weeks	Maintain the MMP	Banerjee et al. (2020)
					Reduce apoptosis	
DM	*Calotropis procera*	Swiss mice	5 mg/kg		Improve IR	De Oliveira et al. (2021)
		HepG2	100 μg/ml	3 h	Increase ETC complex proteins	
DM	*Trigonella foenum-graecum*	3T3-L1 preadipocytes	0–100 μM	48 h	Enhance mitochondrial function	Luan et al. (2018)
					Protect mtDNA	
					Activate the AKT/AMPK pathway	
DM	*Epimedium*	Wistar rats	10 mg/kg	12 weeks	Reduce mitochondrial autophagy	Zhang et al. (2020)
DM	*Ophiocordyceps sinensis*	HUVECs	200 mM	24 h	Reduce mitophagy	Ku et al. (2021)
DM	*Salvia miltiorrhiza*	SD rats	50, 200 mg/kg	7 weeks	Inhibit the NOX4 expression	Zhao et al. (2019)
DM	*Curcuma*	MIN6 cells	20 μM	1 h	Inhibit ER stress	Cao et al. (2022)
					Reduce apoptosis	
DM	*Silybum marianum* Linn. Gaertn	Mouse podocytes	10 μM	24 h	Inhibit NADPH oxidase activity	Khazim et al. (2013)
		OVE26 mouse	100 mg/kg	6 weeks	Inhibit the NOX4 expression	
Obesity	*Zingiber officinale*	3T3-L1 adipocytes			Protect mtDNA	Lee et al. (2021)
		C57BL mice	40, 80 mg/kg	8 weeks	Activate the AMPK/SIRT pathway	
					Enhance mitochondrial function	
					Inhibit ER stress	
Obesity	*Hibiscus sabdariffa*	3T3-L1 adipocytes		48 h	Activate the AMPK pathway	Herranz-Lopez et al. (2020)
					Reduce mitochondrial autophagy	
Obesity	*Toxicodendron vernicifluum*	ob/ob mice, ob/+ mice	20, 40, 80 mg/kg	8 weeks	Activate the AMPK signaling pathway	Hoang et al. (2021)
					Inhibit ER stress	
NAFLD	*Momordica charantia*	HepG2 cells	5 μg/ml	24 h	Inhibit ER stress	Hsu et al. (2021)

#### 3.1.1 Botanical drugs reduce mitochondrial ROS production

Mitochondria are an important source of ROS in mammals. Molecular oxygen undergoes single-electron reduction through the ETC to form ROS, which is then converted to H_2_O_2_ by SOD in the mitochondrial matrix (Murphy, 2009). This effect is enhanced when cells are under hypoxia, and the ROS produced can cause damage to mitochondrial proteins and DNA (Hamanaka and Chandel, 2010; Juan et al., 2021). Botanical drugs can improve mitochondrial dysfunction and reduce ROS production by maintaining mitochondrial membrane potential (MMP), promoting the expression of mitochondrial respiratory chain complex, and reducing mitochondrial autophagy *in vitro* and *in vivo*.

##### 3.1.1.1 Mitochondrial membrane potential

MMP abnormalities lead to ROS production. Previous studies have shown that maintaining a physiological MMP below 140 mv can reduce ROS production, whereas membrane potential hyperpolarization leads to increased ROS production in mitochondrial respiratory chain complexes Ⅰ and Ⅲ (Kaim and Dimroth, 1999; Starkov and Fiskum, 2003; Liu, 2010). Experiments have shown that botanical drugs can restore MMP and reduce ROS production. For example, plants *Salvia plebeia* and *Pueraria lobata* (Willd.) Ohwi effectively reversed MMP loss, reduced ROS production, and protected vascular endothelial cells (Gao et al., 2016; Qin et al., 2016). *In vitro* studies have shown that *Laurus nobilis* Linn. not only maintains the MMP but also increases insulin sensitivity (Bourebaba et al., 2021). *Tinospora sinensis* maintains MMP while reducing islet B-cell apoptosis (Banerjee et al., 2020).

##### 3.1.1.2 Mitochondrial ETC complex

Mitochondrial ETC complexes exist in the mitochondrial and membrane space (Muller et al., 2004) and play an important role in the formation of ROS. For mitochondrial complex Ⅰ in the mitochondrial matrix, when the NADH/NAD ratio increases, the FMN binding site on complex Ⅰ decreases and superoxide is formed (Murphy, 2009). Mitochondrial complex Ⅲ is located in the inner mitochondrial membrane, which produces superoxide through the Q cycle (Hamanaka and Chandel, 2010). *In vitro* and *in vivo* studies have shown that botanical drugs can improve ROS production by regulating the expression of mitochondrial respiratory chain complexes. For example, latex protein from *Calotropis procera* increased the expression of mitochondrial complexes I, III, and V; improved glucose tolerance; and inhibited hepatic glucose production in mice. This makes it a potential agent for the treatment of type 2 DM (De Oliveira et al., 2021).

##### 3.1.1.3 Mitochondrial DNA

MtDNA damage is closely related to mutation and ROS production (Passos et al., 2007). *In vitro* and *in vivo* studies have found that *Trigonella foenum-graecum* seeds can protect the mtDNA of 3T3-L1 adipocytes from the damage caused by ROS induced by high glucose *in vitro* (Luan et al., 2018). *Zingiber officinale Roscoe*’s steamed ginger extract can enhance the mtDNA content and increase thermogenesis to reduce the body weight of HFD-fed mice, which has a certain effect on improving obesity (Lee et al., 2021).

##### 3.1.1.4 Mitochondrial autophagy

Excessive accumulation of ROS leads to mitochondrial autophagy (Schofield and Schafer, 2021). Studies have shown that botanical drugs can maintain mitochondrial function by reducing mitophagy. For example, *Epimedium* and *Cordyceps sinensis* reduce mitophagy *in vivo* (Zhang et al., 2020; Ku et al., 2021), and *Hibiscus hibiscus* increased mitochondrial mass and content of various cell types, activated AMPK pathway, reduced mitochondrial autophagy and alleviated OS induced by glucose lipid toxicity (Herranz-Lopez et al., 2020).

In addition, mitochondrial phosphorylation is an important mechanism for mitochondrial energy production, and electron transport and ATP formation in mitochondrial ETC are related to mitochondrial phosphorylation (Foo et al., 2022). Some botanical drugs have the effect of regulating mitochondrial phosphorylation. As mentioned earlier, the plant *L. nobilis* Linn. plays a role in regulating mitochondrial phosphorylation and MMP (Bourebaba et al., 2021). However, there is a close relationship between the regulation of mitochondrial dysfunction and mitochondrial phosphorylation in plants. However, few studies have focused on the effect of botanical drugs on mitochondrial phosphorylation separately.

#### 3.1.2 Botanical drugs downregulated NADPH expression to reduce ROS production

NADPH oxidase is another major source of ROS. NOX enzymes are membrane-associated proteins that transfer electrons across biofilms, allowing molecular oxygen to accept electrons to form ROS (Bedard and Krause, 2007). The effect of botanical drugs on NOX enzymes is more common in AS. As demonstrated *in vitro*, treatment of HUVECs with *Astragalus mongholicus* can reduce the activity of the NOX enzyme and the level of ROS under oxLDL conditions (Zhu et al., 2019). *In vivo* studies have found that *Lindera obtusiloba* extract can reduce the expression of NOX oxidase subunits p22phox and p47phox and inhibit NOX activity (Ihm et al., 2021). *Prosopis strombulifera*, *Tessaria absinthioides* (Quesada et al., 2021), and *Salvia miltiorrhiza* significantly inhibited the expression of NOX2 and NOX4 (Zhao W. W. et al., 2017; Zhao et al., 2019), while reducing the activation of NF-κB and the expression of vascular adhesion factors and protecting vascular endothelial cells to play an anti-AS role. Regarding DN, extracts from *Silybum marianum* downregulated the activity of the NOX enzyme and reduced ROS production, which played a protective role in podocytes (Khazim et al., 2013).

#### 3.1.3 Botanical drugs alleviate ER stress to reduce ROS production

The accumulation of unfolded proteins in the ER has been termed ER stress (Gardner and Walter, 2011). On the one hand, ER stress can produce ROS through NOX4 and microsomal monooxygenase systems (Zeeshan et al., 2016). On the other hand, ER stress can aggravate mitochondrial dysfunction and produce ROS by regulating Ca ions (Bhandary et al., 2012).

*In vitro* and *in vivo* experiments showed that *Z. officinale* and *Toxicodendron vernicifluum* could reduce ROS production through mTOR/SREBP1/ER stress and then improve fat metabolism to reduce body weight in high-fat diet mice (Hoang et al., 2021; Lee et al., 2021). *Curcuma* can alleviate ER stress and reduce MIN6 cell apoptosis by interfering with the PERK/CHOP pathway (Cao et al., 2022). In addition, *Momordica charantia* also has anti-ER stress and reduces ROS generation (Hsu et al., 2021).

In addition to the aforementioned mechanisms, some botanical drugs can play a role in alleviating AS by regulating the LOX enzyme, such as *S. miltiorrhiza* (Liu et al., 2015) and *Tripterygium wilfordii* Hook F. (Gu et al., 2013).

### 3.2 Scavenging effect of botanical drugs on ROS

Antioxidant enzymes are an important mechanism for the body to cope with the excessive accumulation of ROS. Botanical drugs can play an anti-ROS effect by activating antioxidant enzymes. At the same time, they can play an anti-ROS effect via their reaction with ROS to contribute to alleviating glycolipid metabolic diseases (Table 3).

**TABLE 3:** Research on the removal of ROS by botanical drugs.

Disease	Source	Animals/cell lines	Dose	Duration	Detail	Ref.
DM	*Homalium zeylanicum*	Wistar rats	300, 400 mg/kg	4 weeks	Enhance the activity of antioxidant enzymes	Rout et al. (2020)
DM	*Padina pavonia*	Wistar Rats	50, 100, 200 mg/kg	4 weeks	Enhance the activity of antioxidant enzymes	Germoush et al. (2020)
					Inhibit PPAR-γ pathway	
DN	*Inonotus obliquus*	HGMCs		24 h	Enhance the activity of SOD and CAT	Li et al. (2021b)
DM	*Artemisia caruifolia*	Wistar rats	400 mg/kg	30 days	Enhance the activity of antioxidant enzymes	Sekiou et al. (2021)
DM	*Parkia biglobosa*	Sprague–Dawley rats	200, 400 mg/kg	4 weeks	Enhance the activity of SOD, CAT, and GPX	Ogunyinka et al. (2019)
DM	*Picrorhiza kurroa*	Wistar rats	16 mg/kg	8 weeks	Enhance the activity of SOD, CAT, and GPX	Gimenes et al. (2018)
DM	*Annickia polycarpa*	Wistar rats	20, 100, 500 mg/kg	4 weeks	Clear DPPH free radicals	Lartey et al. (2021)
DM	*Eugenia jambolana*	RAW264.7 cells	10 μM	1 h	Clear ROS	Liu et al. (2018)
DM	*Galega officinalis Smallanthus sonchifolius*	Wistar rats	600, 1,200 mg/kg	14 days	Clear ROS	Hachkova et al. (2021)
DM	*Rumex dentatus* L.	Wistar rats	50, 100, 200 mg/kg	4 weeks	Increase cell sensitivity to insulin	Elsayed et al. (2020)
					Clear ROS	
					Reduce hyperglycemia	
					Enhance the activity of antioxidant enzymes	
DM	*Enicostemma littorale* Blume	Mice	2 mg/ml	2 h	Counteract inflammatory cytokines Oxidative stress-mediated cytotoxicity	Srivastava et al. (2016)
DM	*Hypoxis hemerocallidea*	Wistar rat	200, 800 mg/kg	6 weeks	Antihyperglycemic and antioxidant	Oguntibeju et al. (2016)
DM	*Amaranthus viridis*	Wistar rat	250 mg/kg	6 weeks	Antidiabetic and antioxidant properties	Omodanisi et al. (2017)
AS	*Strawberry*	HepG2	0–1 mg/ml	24, 48, 72 h	Enhance the activity of antioxidant enzymes	Forbes-Hernández et al. (2017)
	*Alnus firma*	3T3-L1 preadipocytes	25, 50, 100 μg/ml		Remove ROS produced during adipose differentiation	Choi et al. (2018)
	*Lannea coromandelica*	RAW264.7 cells Skin Fibroblast cells		1 h	Enhance antioxidant enzyme activity	Alam et al. (2017)
					Activate the Nrf-2 pathway	
					Enhance the expression of antioxidant enzymes	
AS	*Moringa oleifera*	Rabbits	100, 200 mg/kg	4 weeks	Increases antioxidants in plasma	Salvamani et al. (2016)
AS	*Cassia auriculata Linn*	Rats	150, 300, 450 mg/kg	14 days	Investigate antihyperlipidemic	Vijayaraj et al. (2013)
					Antioxidative effect	

#### 3.2.1 Botanical drugs activate antioxidant enzymes to reduce ROS accumulation

Antioxidant enzymes are located in the middle of one of the three layers of antioxidant defense (Lei et al., 2016). These enzymes include SOD, CAT, and GPX. The SOD is a group of metal-containing enzymes that play a crucial antioxidant role in human health. ROS can be converted into H_2_O_2_ under the action of the SOD (Buettner, 2011), which is then decomposed into water by CAT and GPX (Kirkman and Gaetani, 2007; Bhabak and Mugesh, 2010).

Studies have shown that it is effective and feasible to intervene in OS-related diseases with antioxidant enzymes as the key point. For example, the use of oral antioxidant enzymes can enhance ROS clearance and reduce inflammatory responses (Zeng et al., 2021). Treating T2DM rats with *Homalium zeylanicum* and *Padina pavonia* can enhance the activity of antioxidant enzymes, reduce ROS production, and protect islet B cells (Germoush et al., 2020; Rout et al., 2020). *In vivo* experiments have found that *Enicostemma littorale* Blume can enhance the activity of antioxidant enzymes, reduce ROS production, and treat DM (Srivastava et al., 2016). Moreover, some botanical drugs can enhance the activity and expression of antioxidant enzymes and treat DM-related complications (Mihailovic et al., 2021). For example, *Inonotus obliquus* and *Artemisia caruifolia* can improve diabetic kidney damage (Li Y. et al., 2021; Sekiou et al., 2021); *Parkia biglobosa* seeds and *Picrorhiza kurroa* could improve heart damage in DM mice; and the activities of GPX, SOD, and CAT were higher after their intervention (Gimenes et al., 2018; Ogunyinka et al., 2019). The CAT was significantly increased in *Hypoxis hemerocallidea* diabetic rats, which showed anti-hyperglycemic and antioxidant effects (Oguntibeju et al., 2016). All these indicate that botanical drugs play a protective role against DM nephropathy (DN) or heart disease by interfering with antioxidant enzymes.

In AS, *in vitro* experiments found that the *Romina strawberry* variety (AN99.78.51), which is commonly consumed, can activate antioxidant enzymes and damage HepG2 cells (Forbes-Hernandez et al., 2017). Plants such as *Amaranthus viridis* and *Moringa oleifera* also showed excellent enhancement of antioxidant enzyme activity *in vivo* (Salvamani et al., 2016; Omodanisi et al., 2017). *In vivo* experiments found that the flower extract of *Cassia auriculata* could improve hyperlipidemia and the activity of various antioxidant enzymes without obvious adverse reactions (Vijayaraj et al., 2013).

#### 3.2.2 Direct scavenging effect of botanical drugs on ROS

Antioxidants can directly or indirectly inhibit cell damage caused by OS. Antioxidants scour ROS by providing hydrogen or electron antioxidants, and ROS and reactive nitrogen are thought to be a direct pathway (Dinkova-Kostova and Talalay, 2008). Polyphenol extracts from plants have obvious advantages in the removal of ROS and play a role by removing chelating metal ions of ROS (Yahfoufi et al., 2018). For example, *Alnus firma* can eliminate ROS produced during fat accumulation and adipose differentiation in 3T3-L1 cells (Choi et al., 2020), and *Rumex dentatus* L. can improve OS in DM rats by scavenging ROS (Elsayed et al., 2020). In addition, other extracts have a significant free radical scavenging ability; for example, *in vitro* experiments found that *Lannea coromandelica* bark extract (Alam et al., 2017), *Annickia polycarpa* (Lartey et al., 2021), *Eugenia jambolana*, *Galega officinalis*, and *Smallanthus sonchifolius* also had significant ROS clearance ability (Liu et al., 2018; Hachkova et al., 2021).

ROS is a key factor in the OS. Botanical drugs can regulate OS by regulating ROS generation and clearance so that ROS levels tend to be balanced. At the same time, this effect can also play a role by indirectly regulating the up–down pathways related to ROS, which will be described as follows.

### 3.3 Upstream and downstream regulation related to ROS by botanical drugs

**FIGURE 4:**
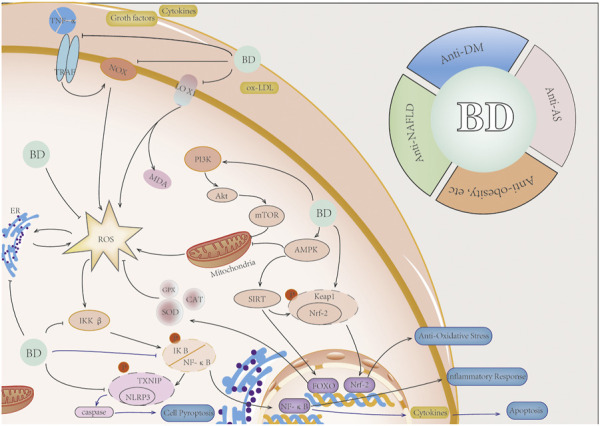
Botanical drugs regulate ROS-related pathways and corresponding anti-inflammatory effects. Botanical drugs can regulate mitochondrial function and reduce ROS production by regulating P13K/AKT/mTOR pathway. Botanical drugs can regulate TNF-α, cytokines, growth factors, and other signaling molecules and reduce the generation of ROS. Botanical drugs can regulate the AMPK pathway, improve mitochondrial energy metabolism, and reduce ROS production. Botanical drugs can regulate the Nrf-2 pathway, activate FOXO and other genes, and increase the expression of antioxidant enzyme genes. Botanical drugs can reduce ROS-induced apoptosis and caspase activation-induced pyroptosis by inhibiting the NF-κB/NLRP3 pathway. Botanical drugs eventually played a role in anti-oxidation, anti-inflammation, anti-apoptosis, and anti-pyroptosis. It has a therapeutic effect on NAFLD, DM, AS, and obesity, among others*.*

Botanical drugs can regulate ROS accumulation and improve the damage caused by ROS accumulation by regulating upstream signals of ROS, such as PI3K signaling pathway and inflammatory factor (tumor necrosis factor) TNF-α, and downstream signals, such as AMPK, NF-κB, and Nrf-2 (Figure 4).

#### 3.3.1 Botanical drugs regulate the upstream signal of ROS and reduce the accumulation of ROS

Botanical drugs can play a regulatory role in regulating ROS by regulating PI3K/AKT pathway and inflammatory factors (Table 4).

**TABLE 4:** Botanical drugs regulate upstream signal transduction pathways related to ROS.

Disease	Source	Animals/cell lines	Dose	Duration	Detail	Ref.
AS	*Herba Erigerontis*	AS rats	6.25, 25 mg/kg	12 weeks	Regulate the Hippo-FOXO3A, PI3K/AKT pathways	Fu et al. (2019)
		HAECs	50, 100, 200 μmol/L	24 h	Inhibit endothelial cell injury and apoptosis	
AS	*Salvia miltiorrhiza* Bunge	HUVECs	10 nM	1 h	Inhibit TNF-α-induced NOX4 expression	Zhao et al. (2017b)
					Inhibit TNF-α-induced NF-κB activation	
					Inhibit the ICAM-1 expression	
AS	*Gynura bicolor*	EA.hy926 cells	10, 50, 100 μg/ml	8 h	Reduce the role of TNF-α	Hsieh et al. (2020)
AS	*Lithospermum erythrorhizon* Sieb. et Zucc	EA.hy926 cells	0, 0.25, 0.5, 1 mM	16 h	Shikonin protects endothelial	Huang et al. (2015)
					Suppressing ROS/NF-κB-mediated ICAM-1	
					Upregulate PI3K/AKT/Nrf-2 pathway	
AS	*Tribulus terrester* Linn.	HUVECs	3, 30 μg/ml	1 h	Decrease mRNA expression of AKT and AMPK	Jiang et al. (2016)
					Endothelial protective effects	
AS	*Prunella vulgaris* Linn.	HASMC	0, 10, 50, 250 μg/ml	2 h	Protect HASMCs on cell viability and THP-1 cell adhesion	Park et al. (2013)
					Reduce the NF-κB activation	
DM	*Alpinia officinarum* Hance	HepG2 cells		24 h	Regulate the PI3K/AKT/Nrf-2/GSK3β pathways	Zhang et al. (2022b)
DM	*Trigonella foenum-graecum*	3T3-L1 preadipocytes	0–100 μM	48 h	Reduce the expression of adipokines	Luan et al. (2018)
					Activate the AKT/AMPK	
					Maintain the MMP	
					Protect mtDNA	
DM	*Rubus amabilis*	MIN6 β-cells	25, 50, 75 μg/ml	24 h	Activate the PI3K/AKT/FoxO1	Sun et al. (2020)
DM	*Epimedium*	Wistar rats	10 mg/kg	12 weeks	Reduce mitochondrial autophagy	Zhang et al. (2020)
					Activate the PI3K/AKT/mTOR pathway	
DM	*Catharanthus roseus*	Wistar Rats	20 mg/kg	8 weeks	Lower levels of TNF-α	Goboza et al. (2019)
DM	*Syzygium aqueum* Alston	Wistar rats	100, 200 mg/kg	14 days	Activate the Nrf-2 pathway	Mahmoud et al. (2021a)
					Reduce TLR-4 activation	
					Reduce pancreatic islet B cell damage	
DM	*Ginkgo biloba* Linn.	HAEC	100 μg/ml	18 h	Reduce endothelial adhesion	Tsai et al. (2013)

##### 3.3.1.1 The PI3K/AKT pathway

Studies have shown that the PI3K/AKT pathway is correlated with ROS, and activated AKT can reduce the production of ROS caused by ischemia (Chatterjee et al., 2012). The mechanism is related to the regulation of mitochondrial function and NOX enzyme by the PI3K/AKT (Stiles, 2009; Nakanishi et al., 2014).

In recent studies, treatment targeting the PI3K/AKT pathway has a certain effect on glycolipid metabolic diseases, such as obesity and DM (Huang et al., 2018). Moderate pharmacological inhibition of the PI3K could be a therapeutic strategy for obesity and metabolic syndrome (Vanhaesebroeck et al., 2021). Moreover, PI3K/AKT is the main pathway of botanical drugs regulating OS-related glycolipid metabolic disorders. For example, botanical drugs can improve DN by regulating the PI3K pathway (Tang et al., 2021). *In vitro* experiments have shown that *Alpinia officinarum* Hance can improve the IR of HepG2 cells through the PI3K/AKT pathway (Zhang X. G. et al., 2022). *Trigonella foenum-graecum* seeds restored dexamethasone-induced glucose uptake in IR3T3-L1 cells by activating the AKT and AMPK (Luan et al., 2018). *Rubus amabilis* active ingredients treat MINI6*β* cells to regulate the activation of the AKT/FoxO1 pathway and play an anti-apoptotic role (Sun et al., 2020). *Epimedium* can reduce mitochondrial autophagy of cavernous smooth muscle cells through PI3K/AKT/mTOR pathway and improve glucose metabolism and ROS production in T2DM rats with erectile dysfunction (Zhang et al., 2020).

For AS, *in vivo* experiments showed that *Herba Erigerontis* could upregulate the expression of Hipo/FoxO3a and PI3K/AKT and reduce the occurrence of OS in vascular endothelial cells in high-fat diet rats (Fu et al., 2019). *In vitro*, *S. miltiorrhiza* Bunge has been found to reduce the production of intracellular reactive oxygen species through the PI3K/Akt/MEK1/Nrf-2 pathway and has therapeutic effects on vascular diseases (Lee et al., 2012). *Lithospermum erythrorhizon* and *Tribulus terrestris* can inhibit OS and protect vascular endothelial cells by activating the PI3K/AKT pathway and inhibiting the NF-κB pathway, and *L. erythrorhizon* Sieb. et Zucc*.* can also inhibit the NF-κB pathway activation and improve inflammation (Huang et al., 2015; Jiang et al., 2016). *Ginkgo biloba* Linn*.* extract can reduce ROS production by activating the AKT/eNOS and play a protective role in endothelial cells *in vitro* (Tsai et al., 2013).

Botanical drugs can reduce the excessive production of ROS; can improve IR, vascular endothelial cell injury, and islet B-cell injury by regulating the PI3K/AKT pathway; and have certain therapeutic effects on DM, AS, NAFLD, and other diseases.

##### 3.3.1.2 TNF-α and other inflammatory factors

ROS production can be stimulated by signal molecules, such as growth factors, cytokines, and circulating exosomes (Sies and Jones, 2020). For example, TNF-α, circular RNA, and interleukin-4 induce ROS production (Lee et al., 2005; Sharma et al., 2008; Saaoud et al., 2021). Meanwhile, the crosstalk between inflammation and OS makes inflammatory factors, other information molecules, and ROS have inextricable relationships (Forrester et al., 2018). All these provide a rationale for reducing ROS production by inhibiting the expression of inflammatory factors and cytokines.

Previous studies have shown that botanical drugs have significant regulatory effects on signaling molecules, such as inflammatory factors in glucolipid metabolism diseases. *In vitro* and *in vivo* experiments have shown that botanical drugs can inhibit the overexpression of TNF, vascular adhesion factor (VCAM), and cytokines; reduce ROS generation and vascular endothelial damage caused by them; and have certain therapeutic effects on AS (Gu et al., 2013; Caliceti et al., 2017; Su et al., 2018). TNF-α can activate NOX4 to induce ROS generation, and *S. miltiorrhiza* can inhibit TNF-α-induced ROS generation through hydrogen bond interaction with NOX4, the production of inflammatory factors, and the release of VCAM-1 (Zhao W. W. et al., 2017). *Gynura bicolor* can reduce the effects of TNF-α in several ways (Goboza et al., 2019), and *Catharanthus roseus* can reduce TNF-α levels to reduce ROS (Hsieh et al., 2020). *In vitro* experiments of *Prunella vulgaris* and *in vivo* experiment of *Ligusticum chuanxiong* Hort. showed that both plants suppressed OS and reduced inflammation in human vascular smooth muscle cells following TNF-α treatment (Park et al., 2013; Li et al., 2014). In addition, botanical drugs can interfere with the activity of TNF-α receptor TRAF to interfere with OS. For example, *Syzygium aqueum* Alston was found to reduce the damage of OS and inflammation on islet B cells by downregulating the expression level of the TRAF6, NF-κB-mediated inflammation, and the effect of TNF-α (Mahmoud et al., 2021a). In addition, berberine and *S. miltiorrhiza* extracts can attenuate TNF-α-induced phosphorylation of IkBα, reduce inflammation and OS, and protect vascular endothelial cells (Zhao W. W. et al., 2017; Caliceti et al., 2017).

#### 3.3.2 Botanical drugs regulate the downstream pathways of ROS and reduce the accumulation of ROS

Botanical drugs can regulate ROS downstream pathways (e.g., the AMPK pathway, the NF-κB/NLRP3 inflammasome pathway, and the Nrf-2 pathway) to reduce cell damage caused by excessive ROS, regulate inflammation, or activate the expression of antioxidant enzymes (Table 5).

**TABLE 5:** Botanical drugs regulate the downstream signal transduction pathways related to ROS.

Disease	Source	Animals/cell lines	Dose	Duration	Detail	Ref.
Obesity	*Zingiber officinale*	C57BL/6 mice	40, 80 mg/kg	8 weeks	Activate the AMPK pathway	Lee et al. (2021)
					Enhance mitochondrial function	
					Inhibit ER stress	
Obesity	*Toxicodendron vernicifluum*	ob/ob mice, ob/+ mice	20, 40, 80 mg/kg	8 weeks	Activate the AMPK pathway	Hoang et al. (2021)
					Inhibit ER stress	
Obesity	*Rubia tinctorum Rubia cordifolia*	3T3-L1 cells	50, 100μM	48 h	Activate the AMPK pathway	Nam et al. (2019)
		C57BL/6 mice	40, 80 mg/kg	10 weeks		
Obesity	*Hibiscus sabdariffa*	3T3-L1 adipocytes		48 h	Activate the AMPK pathway	Herranz-Lopez et al. (2020)
					Reduce mitochondrial autophagy	
NAFLD	*Dillenia indica*	HepG2 cells	5, 10 μg/ml	2 h	Activate the SIRT-1/AMPK pathway	Poornima et al. (2022)
NASH	*Alisma plantago-aquatica* subsp*. orientale*	C57BL/6 mice	15, 30, 60 mg/kg	4 weeks	Regulate autophagy	Wu et al. (2018)
		WRL-68 cells, LX2 cells	1-16 μmol/L	48 h	Activate the AMPK pathway	
NAFLD	*Antrodia Cinnamomea*	HepG2 cells			Inhibit the NLRP3 inflammasome activation	Yen et al. (2020)
		RAW264.7 cells			Inhibit ER stress	
		C57BL/6 mice	100 mg/kg	10 days	Inhibit the NLRP3 inflammasome activation	
NAFLD	*Artemisia capillaris*	FL83B hepatocytes	25–200 μM	24 h	Inhibit apoptosis	Li et al. (2021a)
					Inhibit the NLRP3 inflammasome activation	
		C57BL/6J mice	25, 50, 100 μmol/kg	5 weeks	Inhibit the NLRP3 inflammasome activation	
NAFLD	*Juglans regia*	SPF-grade	300 mg/kg	12 weeks	Inhibit the NLRP3 inflammasome activation	Fang et al. (2022)
NAFLD	*Cannabis sativa*	C57B/6J mice	3 mg/kg	16 weeks	Inhibit the activation of the NF-κB pathway	Jiang et al. (2016)
					Inhibit the NLRP3 inflammasome activation	
					Inhibit pyroptosis	
NAFLD	*Ilex chinensis* Sims	Larval zebrafish	10, 20, 40 μM	13 days	Reduce inflammation	Deng et al. (2021)
		HepG2 cells	10, 15, 20 μM	24 h	Activate the Keap1/Nrf-2 pathway	
	*Trigonella foenum-graecum*	3T3-L1 Preadipocytes	0–100 μM	48 h	Enhance mitochondrial function	Luan et al. (2018)
					Protect mtDNA	
					Activate the AKT/AMPK pathways	
DM	*Trigonella foenum-graecum*	3T3-L1 Preadipocytes			Activate the AKT/AMPK pathways	Li et al. (2018)
DM	*Premna herbacea*	L6 muscle cells	0.5–10 µM	6 h	Activate the JNK/AKT/mTOR pathway	Kashyap et al. (2021)
		SD rats	250 mg/kg		Improve IR	
DM	*Anoectochilus roxburghii*	HUVECs	10, 20, 30 μg/ml	1 h	Inhibit the NF-κB pathway	Liu et al. (2017)
		ICR mice	100, 300 mg/kg	15 days		
DM	*Cistanche tubulosa*	LC-540 cells	50 μL/ml	24 h	Inhibit the NF-κB pathway	Kong et al. (2018)
		Sprague–Dawley rats	160, 320 mg/kg	6 weeks	Enhance the activity of antioxidant enzymes	
DM	*Ganoderma lucidum*	INS-1 cells	0–200 μg/ml	4 h	Inhibit NF-κB pathway	Liang et al. (2020)
DN	*Inonotus obliquus*	Human glomerular mesangial cells			Activate Nrf-2 pathway	Li et al. (2021b)
DM	*Hydrangea paniculata*	Wistar rats	15, 30, 45 mg/kg	3 months	Activate the Nrf-2 pathway	Sen et al. (2019)
DM	*Ginkgo biloba*	Wistar rats	20 mg/kg	12 weeks	Activate the Nrf-2 pathway	Su et al. (2022)
DN	*Ginkgo biloba*	DBA/2 mice	50, 200 mg/kg	4 weeks	Activate the Nrf-2/HO-1 pathway	Chang et al. (2021)
DM	*Scutellaria baicalensis*	HUVECs and HAOECs	50 μM	72 h	Activate the Nrf-2 pathway	Chen et al. (2019)
		C57BL/6 mice	50 mg/kg	4 weeks	Reduce endothelial cell apoptosis	
DM	*Anoectochilus roxburghii* Wall. Lindl.	HUVECs	10–30 μg/ml	48 h	Inhibit the expression of RAGE	Liu et al. (2016)
					Decrease intracellular ROS generation	
DM	*Paeonia lactiflora* Pall.	RSC96 cells	1, 10, 100 μM	48 h	Decrease ROS and MDA levels	Yang et al. (2016)
					Increasing GST and GPX activity	
AS	*Cnidium monnieri*, *Angelica gigas Nakai*	HUVECs		30 min	Attenuate vascular inflammation	Choi et al. (2018)
					Activate the Nrf-2/HO-1 pathway	
AS	*Salvia plebeia*	HUVECs	0.1, 1, 10 μM	24 h	Inhibit the NLRP3 inflammasome activation	Qin et al. (2016)
					Inhibit the NF-κB pathway	
AS	*Sophora flavescens*	HUVECs	2, 4, 8 µM	1 h	Activate the SIRT1/Nrf-2 pathway	Jin et al. (2021b)
					Inhibit the NLRP3 inflammasome activation	
					Inhibit the pyroptosis	
AS	*Crataegus aronia*	Wistar rats	200 mg/kg	4, 8 weeks	Inhibit the NLRP3 inflammasome activation	Shatoor and Al Humayed (2020)
AS	*Ligusticum chuanxiong* Hort.	Sprague–Dawley rats	600 mg/kg	12 weeks	Improve serum lipid profiles	Li et al. (2014)
					Reduce the ROS level	
AS	*Scutellaria baicalensis* Georgi	HUVECs	2, 5, 10 μM	6 h	Prevent atherosclerotic lesions	Ku and Bae (2015)
		Mice	2, 5, 10 μM			
AS	*Rubus coreanus* Miq.	RAW264.7 cells		4 h	Improve plasma lipid profile	Kim et al. (2013)
		C57BL/6 J mice	1.67 g/kg	14 weeks	Inhibit inflammation-associated gene	
					Inhibit phase II enzyme function reduces	
AS	*Panax ginseng* C. A. Meyer	Wistar rats	100 mg/kg	10 weeks	Protected endothelial cells	Fan et al. (2016)
					Activate the Nrf-2 pathway	

##### 3.3.2.1 The AMPK pathway

The AMPK pathway is an important regulator of energy metabolism in the body, which can regulate substance metabolism and synthesis and induce autophagy (Zhao Y. et al., 2017). Currently, all mechanisms by which the AMPK is activated by ROS are not fully understood (Ren and Shen, 2019). However, it has been shown that exogenous or glucose oxidase-generated H_2_O_2_ induces direct S-glutathionylation of cysteine residues Cys299 and Cys304 on the AMPKα subunit. It has been demonstrated that ROS can directly activate the AMPK pathway (Zmijewski et al., 2010).

Botanical drugs have a certain effect on improving lipid metabolic diseases by regulating the AMPK pathway. For example, the use of *T. vernicifluum* and *Z. officinale* extracts can effectively reduce ROS levels and highly maintain the AMPK/SIRT1 signaling, and *Z. officinale* extracts could inhibit hepatic dyslipidemia and regulate lipid metabolism (Hoang et al., 2021; Lee et al., 2021). *In vitro* and *in vivo* experiments have found that purpurin derived from *Rubia tinctorum* L. and *Rubia cordifolia* can exert anti-obesity effects through the AMPK pathway (Nam et al., 2019). For NAFLD, *Dillenia indica* leaf and *Alisol A 24-acetate* treatment significantly increased the expression of the AMPK/SIRT pathway, reduced ROS production, and had a certain protective effect on hepatocytes (Wu et al., 2018; Poornima et al., 2022).

Moreover, the intervention of AMPK has significant benefits in improving DM. *Trigonella foenum-graecum* reduces the incidence of IR and restores glucose uptake and insulin sensitivity in adipocytes by activating the AKT and AMPK, which are inhibited by dexamethasone. Phosphorylation of AKT/AMPK induced by *Premna herbacea* extract activates AS106 cells and increases glucose uptake by muscle cells (Li et al., 2018; Luan et al., 2018; Kashyap et al., 2021). *In vivo*, *Aspalathus linearis* was found to increase AMPK phosphorylation, reduce intracellular ROS production in islet B cells, and increase glucose uptake in myocytes (Kamakura et al., 2015). In addition, some botanical drugs can regulate the expression of adipogenic genes, such as PPAR and SREBP-1c, and reduce the production of inflammatory factors while regulating OS and AMPK (Herranz-Lopez et al., 2020; Lee et al., 2021).

##### 3.3.2.2 The NF-κB pathway

NF-κB is an intracellular transcription factor that plays an important role in immunity and inflammation (Vallabhapurapu and Karin, 2009). Studies have shown that high glucose induces the production of ROS and stimulates the phosphorylation of IKKβ and NF-κB p65, but the phosphorylation of IKKβ is inhibited when ROS inhibitors are used, suggesting that ROS can promote the phosphorylation of IKKβ/NF-κB (Qin et al., 2016). Moreover, H_2_O_2_ can also directly regulate NF-κB (Sies and Jones, 2020).

In AS, NAFLD, and obesity, typical disorders of lipid metabolism, botanical drugs attenuate ROS and inflammation and reduce cell damage and apoptosis by regulating the NF-κB pathway. For example, *in vitro* experiments showed that pretreatment of HUVECs with *Cnidium monnieri* and *Angelica gigas Nakai* inhibited the NF-κB nuclear displacement caused by TNF-α stimulation and downregulated VACM expression and ROS generation (Choi et al., 2018). *In vitro* and *in vivo* experiments have found that the fruits of *Scutellaria baicalensis* Georgi and *Rubus coreanus* Miq. can reduce vascular inflammation by inhibiting ROS production and the NF-κB pathway and have a therapeutic effect on AS (Kim et al., 2013; Ku and Bae, 2015). *Anoectochilus roxburghii* and *Cistanche tubulosa* can protect vascular endothelial cells and reproductive function in diabetic rats by inhibiting the NF-κB pathway (Liu et al., 2017; Kong et al., 2018). For DM, *Ganoderma lucidum* can protect islet B cells by inhibiting NF-κB, JNK, and MAPK pathways (Liang et al., 2020). *In vitro* experiments have found that *Anoectochilus roxburghii* inhibits NF-κB to reduce ROS production and has a certain therapeutic effect on diabetic vasculopathy (Liu et al., 2016).

##### 3.3.2.3 The NLRP3 inflammasome

For NLRP3 inflammasome, ROS and NF-κB activate the NLRP3 inflammasome (Tschopp and Schroder, 2010). Studies have shown that ROS can activate NLRP3 inflammasome via TXNIP, and TXNIP/NLRP3 activation is inhibited after ROS clearance (Dan Dunn et al., 2015; Mai et al., 2020). The NLRP3 inflammasome plays an important role in the development of DM, NAFLD, and AS (Hoseini et al., 2018). Excessive activation of NLRP3 will lead to the excessive release of inflammatory factors and pyrosis of cells (Jin et al., 2021a). It has been documented that AS is a key target for treating NAFLD, AS, and DM (Tang et al., 2019; Wu et al., 2021; Yu et al., 2022). Here, it has been proved that the active ingredients of botanical drugs can treat diabetes and its complications through NLRP3, and the botanical drugs and active ingredients are summarized, so we will not elaborate on this (Bai et al., 2021).

Some botanical drugs can target NLRP3 during AS and exert their effects, as found *in vivo*, *S. plebeia* can improve high glucose-mediated endothelial dysfunction by inhibiting PKC*β*II-related NLRP3 inflammasome activation and NF-κB signaling (Qin et al., 2016). *Sophora flavescens* can alleviate the NLRP3-mediated apoptosis in HUVECs stimulated by LDL (Jin et al., 2021b). *Crataegus aronia* inhibited the levels of NLRP3, caspase-1, and mature IL-1β in aortic tissues of high-fat diet rats and reduced the nuclear accumulation of NF-κB. Thus, it plays a role in reducing ROS (Shatoor and Al Humayed, 2020). All of the aforementioned plants can alleviate AS, which is worth exploring in depth. For NAFLD, some botanical drugs can target NLRP3 to exert anti-NAFLD effects, such as *Antrodia Cinnamomea* (Yen et al., 2020), *Artemisia capillaris* (Li B. et al., 2021), *Juglans regia* green husk (Fang et al., 2022), and extracts of *Cannabis sativa* (Jiang et al., 2021), which were found to reduce ROS, inhibit the NLRP3 *in vitro* and *in vivo*, and have a certain therapeutic effect on NAFLD.

##### 3.3.2.4 The Nrf-2 pathway

Nrf-2 is also a nuclear factor in the cytoplasm and an important antioxidant factor in the body. It plays an important role in regulating ROS levels (Kaspar et al., 2009). The genes regulated by Nrf-2 include HO-1, GST, and NQO1, and their expressed enzymes have antioxidant effects (Liu et al., 2007). Current studies have found that targeting Nrf-2 has a clear effect on the treatment of cancer, DM and its complications, and AS (Axelsson et al., 2017; Niu et al., 2019; Sivinski et al., 2021).

In DM and its complications, botanical drugs can regulate the expression of antioxidant enzymes through Nrf-2, be antioxidant and anti-inflammatory, and reduce apoptosis. For example, *in vitro* experiments showed that the Nrf-2 expression was significantly enhanced and ROS was significantly reduced under the treatment of *I. obliquus* and *G. biloba* extract, which improved renal podocyte injury caused by DN (Li Y. et al., 2021; Chang et al., 2021). Similarly, the *G. biloba* extract reduced retinal damage in diabetic rats by activating Nrf-2 *in vivo* (Su et al., 2022). *Hydrangea paniculata* can exert beneficial effects on DN by increasing the Nrf-2 expression and inhibiting TGF-smad signaling activation (Zhang et al., 2019). *In vitro* experiments showed that the ethyl acetate fraction of *Penthorum chinense* Pursh stems could directly bind to the Keap1 protein, resulting in nuclear translocation of Nrf-2 and activation of antioxidant-related proteins (Sun et al., 2021). *Scutellaria baicalensis* root improves hyperglycemia-induced vascular endothelial injury by promoting the Nrf-2 nuclear enrichment (Chen et al., 2019). For DM peripheral neuropathy, *Paeonia lactiflora* Pall. pretreatment of RSC96 cells showed that *P. lactiflora* Pall. could inhibit the ROS production induced by high glucose and reduce the apoptosis of RSC96 cells through Nrf-2 (Yang et al., 2016).

The activation of Nrf-2/HO-1 signaling plays an important role in protecting endothelial cells (Zhang et al., 2021). Botanical drugs can treat AS by interfering with Nrf-2, which can protect vascular endothelial cells, anti-inflammation, and anti-oxidation. For example, when *Panax ginseng* and Ginsenoside Rb1 were used to treat AS rats, serum NO and SOD levels were upregulated, and Nrf-2 nuclear translocation and HO-1 activation were observed. Moreover, Ginsenoside Rb1 inhibited oxLDL-induced p38 and VCAM-1 expression and reduced the adhesion of monocytes to vascular endothelial cells (Fan et al., 2016).

In addition, the intervention of botanical drugs on NAFLD can play an anti-inflammatory and anti-oxidative effect by regulating the expression of Nrf-2 and protecting liver cells. As found in *in vivo* and *in vitro* experiments, intervention with *Ilex Chinensis* can increase the expression of the Nrf-2 and Keap1 genes, reduce ROS production, and have a certain improvement effect on NAFLD of zebrafish larvae (Deng et al., 2021).

### 3.4 Botanical drugs alleviated inflammation, apoptosis, and IR caused by ROS

Abnormal levels of ROS often cause problems other than OS, such as cell apoptosis, IR, and tissue inflammation*.* These pathological conditions interact with OS and aggravate the development of the disease. Botanical drugs can improve these problems caused by ROS abnormalities. Later, we will explain the effects of related drugs from the perspective of regulating ROS and anti-apoptosis and improving IR and anti-inflammation (Table 6).

**TABLE 6:** Botanical drugs alleviated inflammation, apoptosis, and IR caused by ROS.

Disease	Source	Animals/cell lines	Dose	Duration	Detail	Ref.
DM	*Crassocephalum crepidioides*	INS-1	50–1,000 μg/ml	24 h	Reduce apoptosis	Bahar et al. (2017)
		Wistar rats	150, 300 mg/kg	48 h		
DM	*Tinospora sinensis*	Wister rats	100–400 mg/kg	4 weeks	Maintain the MMP	Banerjee et al. (2020)
					Reduce apoptosis	
DM	*Nepeta angustifolia* C. Y. Wu	Sprague–Dawley rats	60, 120, 240 mg/kg	56 days	Inhibit inflammation	Huang et al. (2020)
		HBZY-1 cells		12 h	Reduce apoptosis	
DM	*Herba Epimedii*	db/db mice	10, 20, 40 mg/kg	7 weeks	Reduce inflammatory cytokines	Li et al. (2022)
					Inhibit the NF-κB pathway	
DM	*Alpinia officinarum* Hance	HepG2 cells		24 h	Regulate the PI3K/AKT/Nrf-2/GSK3β pathways	Zhang et al. (2022b)
DM	*Syzygium jambos*	Wistar rats	100, 200 mg/kg	14 days	Lower levels of inflammatory cytokines	Mahmoud et al. (2021b)
DM	*Laurus nobilis* Linn.	HepG2 cells	1 µg/ml	24 h	Reduce total intracellular ROS levels	Bourebaba et al. (2021)
DM	*Paeonia suffruticosa* Andr.	HBZY-1 rat mesangial cells	1.25, 2.5, 5 g/kg	24 h	Attenuate MC on macrophages migration	Zhang et al. (2014)
AS	*Morinda citrifolia*	THP-670 cells	1 ng/ml	2 days	Inhibit inflammation	Ishibashi et al. (2017)
AS	*Echinodorus grandiflorus*	Rabbits	10, 30, 100 mg/kg	60 days	Inhibit inflammation	Gasparotto et al. (2019)
AS	*Dendrobium huoshanense*	Zebrafish	0.1, 1, 10 mg/L	10 days	Inhibit inflammation	Fan et al. (2020)
AS	*Dendrobium officinale*	Zebrafish	0.1, 1, 10 mg/L	45 days	Inhibit inflammation	Han et al. (2021)
		HUVECs	0.1, 1, 10 mg/L	24 h	Inhibit inflammation	

**NOTE:** INS-1: a rat insulinoma cell line.

#### 3.4.1 Botanical drugs regulate ROS-associated apoptosis and pyroptosis

##### 3.4.1.1 Apoptosis

Excessive accumulation of ROS can lead to impaired mitochondrial function, lipid peroxidation, decreased ATP level, and, finally, cell necrosis (Orrenius et al., 2007). Moreover, ROS can induce apoptosis by mediating the oxidation of cardiolipin and promoting the release of cytochrome C from mitochondria. This demonstrates the bridge-like role of ROS between apoptosis, necrosis, and OS (Kaminskyy and Zhivotovsky, 2014). Botanical drugs can play an anti-apoptotic role by regulating the expression of pro- and anti-apoptotic genes. *In vitro* and *in vivo* studies have found that plants *Crassocephalum crepidioides* and *T. sinensis* can exert antioxidant and anti-apoptotic effects and reduce islet B-cell apoptosis, thereby improving DM (Bahar et al., 2017; Banerjee et al., 2020). *Nepeta angustifolia* C. Y. Wu can improve DN through anti-apoptotic effects (Huang et al., 2020).

##### 3.4.1.2 Pyroptosis

Pyroptosis is a novel mode of programmed cell death characterized by the dependence on inflammatory caspases and the formation of activated gasdermin-D pores in the plasma membrane, eventually leading to cell rupture and the release of cytokines (Shi et al., 2017). NLRP3 plays an important role in pyroptosis (Sutterwala et al., 2006), so the regulation of ROS production and the activation of NLRP3 play an important role in anti-pyroptosis. In *in vitro* and *in vivo* studies of NAFLD, berberine has been shown to reduce pyroptosis by regulating the ROS/NLRP3 pathway (Mai et al., 2020).

#### 3.4.2 Botanical drugs alleviated ROS-mediated IR

As discussed previously, the accumulation of ROS will induce IR, and botanical drugs increase cellular insulin sensitivity. In the hyperglycemia environment, *L. nobilis* Linn. extract could improve the decrease in INSR, AKT, and PI3K protein abundance induced by high insulin in HepG2 cells, and it alleviated IR while reducing ROS (Bourebaba et al., 2021). *Premna herbacea* improved IR, enhanced glucose uptake, and reduced ROS production in rat skeletal muscle cells through JNK/AKT/mTOR signaling (Kashyap et al., 2021). For T2DM, *A. officinarum* Hance can improve IR, as indicated by increased glucose uptake and glucose consumption in HepG2 cells, and this effect occurs through the PI3K/AKT/Nrf-2/GSK3β pathway (Zhang X. G. et al., 2022). *Herba Epimedii* alleviates IR by regulating the IRS1/AKT signal transduction pathway in db-/db-mice (Li et al., 2022). At the same time, this corroborates the importance of PI3K/AKT in combating IR (Huang et al., 2018).

#### 3.4.3 The effects of botanical drugs against the inflammation associated with ROS

OS and inflammation play an important role in the development of abnormal glycolipid metabolic disease. ROS can also induce inflammation (Lei et al., 2015), so the regulation of ROS is also reflected in inflammation. *Syzygium jambos* bark extract protected islet B-cell in DM rats through IRS-2/AKT/GLUT4, ameliorated the elevation of TNF-α and IL-10, and exerted a regulatory effect on inflammation and OS (Mahmoud et al., 2021b). *In vivo* experiments have found that *Dracaena cochinchinensis (Lour.) S. C. Chen* and *Moutan Cortex* play anti-inflammatory and anti-oxidative effects by downregulating inflammatory factors and ROS levels and have certain therapeutic effects on DM (Chen et al., 2013; Zhang et al., 2014). *In vivo* and *in vitro* experiments of botanical drugs for the treatment of NAFLD have shown that berberine can control the release of inflammatory factors through ROS/NLRP3, improve the inflammation of NAFLD, protect hepatocytes, and slow the progression of the disease (Mai et al., 2020). In AS, *Morinda citrifolia* (Ishibashi et al., 2017), *Echinodorus grandiflorus* (Gasparotto et al., 2019), *Dendrobium huoshanense* (Fan et al., 2020), and *Dendrobium officinale* (Han et al., 2021) also play an antioxidant and anti-inflammatory role.

## 4 Conclusion and prospects

The incidence of glycolipid metabolic disease is gradually increasing, as well as the number of people affected by it, which forces people to find more ways to treat this kind of disease. As discussed earlier, ROS, as a key part of OS, plays an important role in the development of glycolipid metabolic diseases. Therefore, interfering with ROS to treat lipid metabolic diseases is a feasible and effective means in the future. For example, mitoTEMPOL and Q10 target mitochondria to reduce ROS in order to treat diabetes-related vascular damage (Graham et al., 2009; Dikalova et al., 2010) and nanoparticle drugs target ROS to improve AS (Wang et al., 2018; Zhang Z. et al., 2022) and the application of antioxidants in NAFLD (Ma et al., 2021). With this goal in view, this study summarizes the literature on botanical drugs to improve glycolipid metabolic diseases by regulating ROS from 2013 to 2022. In addition, the application parts, types of active ingredients, and extraction methods of botanical drugs involved were summarized. In addition, the mechanism of the intervention of botanical drugs on ROS to treat glycolipid metabolic diseases is briefly described, hoping this study can provide some help for the clinical use of botanical drugs.

However, there are still many deficiencies in the current research on ROS and glycolipid metabolic diseases. For example, many signaling pathways are related to OS, including but not limited to the signaling pathways discussed previously. In the future, if we continue to explore new pathways related to ROS in glycolipid metabolic diseases, or further study the cross-talk of existing pathways and develop drugs that can intervene in multiple pathways, It is believed that these may be helpful to improve OS in glycolipid metabolic diseases. These are going to be interesting new lines of research.

There are also some drawbacks to the study of botanical drugs. For example, there are more studies on the mechanism of botanical drugs or monomer components but fewer studies on adverse reactions and toxic side effects. There is insufficient clinical trial research and a lack of research on drug metabolism and kinetics. In order to promote the application of botanical drugs in glycolipid metabolic diseases and strengthen the research on the aforementioned problems, the following points are also worth developing: the extraction, processing, and storage of the effective ingredients of botanical drugs. The contents of the same active ingredient in different plants were compared. There is also the combination of a variety of plants, such as the use of herbal decoction and the combination of botanicals and chemical drugs (such as *Erigeron breviscapus* and enalapril to improve diabetic kidney injury; Xu et al., 2013). In addition, plant drugs do not only treat glucose and lipid metabolism diseases by regulating ROS but also include inflammation, apoptosis, cell proliferation, and other mechanisms. Therefore, the study of the combined effects of multiple mechanisms is also a good direction.

In summary, many botanical drugs have shown therapeutic effects on glycolipid metabolic diseases by regulating ROS. However, there are still many deficiencies in the current research. Although botanical drugs are still used empirically in the treatment of glucose and lipid metabolic diseases in many areas, it is obviously of great benefit to the clinical promotion of botanical drugs if a more in-depth study of the mechanism can be carried out. It is believed that better utilization of these widely available, low-cost, and complex botanical drugs will add new and powerful means for the treatment of glycolipid metabolic diseases.

## Figures and Tables

**Table:** 

**AGE**	Advanced glycation
**AMPK**	AMP-activated protein kinase
**AS**	Atherosclerosis
**ATP**	Adenosine triphosphate
**BBR**	Berberine
**DA**	Danshenol A
**DM**	Diabetes mellitus
**DN**	Diabetic nephropathy
**ER**	Endoplasmic reticulum
**ETC**	Electron transport chain
**ETF**	Electron transfer flavoprotein
**FOXO**	Forkhead box O
**GLUT-4**	Glucose transporter-4
**GPX**	Glutathione peroxidase
**GSH**	Glutathione
**H2O2**	Hydrogen peroxide
**HDL**	High-density lipoprotein
**HUVECs**	Human umbilical vein endothelial cells
**ICAM-1**	Intercellular adhesion molecule-1
**IR**	Insulin resistance
**IRS**	Insulin receptor substrate
**JNK**	C-Jun N-terminal kinase
**LDL**	Low-density lipoprotein
**LOE**	*Lindera obtusiloba* extract
**LOX**	Lipoxygenase
**MMP**	Mitochondrial membrane potential
**mtDNA**	Mitochondrial DNA
**mTOR**	Mammalian target of rapamycin
**NADPH**	Nicotinamide adenine dinucleotide phosphate hydrogen
**NAFLD**	Non-alcoholic fatty liver disease
**NOX**	Nicotinamide adenine dinucleotide phosphate hydrogen oxidases
**O2-**	Superoxide
**OS**	Oxidative stress
**PARP**	Polymerase
**PH**	Hydrogen ion concentration
**PI3K**	Phosphatidylinositol 3-kinases
**PKC**	Protein kinase c
**REDOX**	Reduction and oxidation
**ROS**	Radical oxygen species
**SOD**	Superoxide dismutase
**TNF**	Tumor necrosis factor
**VCAM-1**	Vascular cell adhesion molecule-1
**VLDL**	Very low-density lipoprotein
**VSMC**	Vascular smooth muscle cell
